# Clinical heterogeneity and potential high pathogenicity of the Mmalton Alpha 1 antitrypsin allele at the homozygous, compound heterozygous and heterozygous states

**DOI:** 10.1186/s13023-015-0350-6

**Published:** 2015-10-07

**Authors:** Philippe Joly, Olivier Guillaud, Valérie Hervieu, Alain Francina, Jean-François Mornex, Colette Chapuis-Cellier

**Affiliations:** Unité de Pathologie Moléculaire du Globule Rouge, Laboratoire de Biochimie et de Biologie Moléculaire, Hôpital Edouard Herriot, Hospices Civils de Lyon, Lyon, France; Service d’anatomie pathologique, Hôpital Edouard Herriot, Hospices Civils de Lyon, Lyon, France; Service d’hépato-gastroentérologie, Hôpital Edouard Herriot, Hospices Civils de Lyon, Lyon, France; Laboratoire d’Immunologie, Centre de Biologie Sud, Centre hospitalier Lyon-Sud, Hospices Civils & Université Claude Bernard-Lyon 1, Lyon, France; Service de pneumologie, Groupement Hospitalier Est, Hospices Civils & Université Claude Bernard-Lyon 1, Bron, France; Centre de Recherche et d’Innovation sur le Sport (CRIS)—EA 647, Université Claude-Bernard Lyon 1, Villeurbanne, France; Labex GR-Ex, Institut Universitaire de France, Paris, France

## Abstract

**Background:**

Alpha 1 antitrypsin (A1AT) deficiency (A1ATD) is potentially associated with a high degree of liver and/or lung disease. Apart from the most frequent deficiency alleles, Pi S and Pi *Z*, some A1AT alleles of clinical significance may be easily misdiagnosed. This is typically the case of the Pi Mmalton variant which shares the same ‘gain-of-function’ liver toxicity than Pi Z and the same ‘loss of function’ lung disease as well.

**Methods:**

The biological diagnosis of A1ATD typically relies on a low serum concentration associated with an abnormal isoelectric focusing (IEF) pattern of migration. However, Sanger direct DNA sequencing may be required for deficiency alleles without biochemical expression (Null alleles) or for A1AT variants whose IEF profiles resemble the wild-type and sub-types M allele but with a low concentration.

**Results:**

We report four cases of A1ATD involving the deficient Pi Mmalton allele with very different clinical expressions: (i) one Mmalton/Mmalton with liver fibrosis and cirrhosis, (ii) two Mmalton/Z with chronic pulmonary obstructive disease in one case and (iii) one M/Mmalton without liver or lung disease. In both cases, the correct diagnosis has necessitated a genetic analysis.

**Conclusions:**

Our study provides another example of Pi Mmalton homozygosity associated with a severe liver disease that emphasizes the necessity of a not delayed diagnosis. The great clinical heterogeneity of the other genotypes (which is in agreement with the literature data) questions about the role of environmental and other modifier genes in the pathogenicity of A1ATD.

## Background

Alpha 1 antitrypsin (A1AT) deficiency (A1ATD) is a common autosomal co-dominant genetic disorder caused by mutations on the *SERPINA1* gene (chromosome 14) that may be clinically characterized by lung (emphysema, chronic obstructive pulmonary disease-COPD) and/or liver (cirrhosis) disease. These two different clinical expressions reflect the two pathophysiological pathways of A1ATD which are respectively designated as the ‘loss-of-function’ and ‘gain-of-function’ mechanisms [1]. In the first one, low A1AT plasma concentrations are responsible for the pulmonary parenchyma destruction since the local degradation of neutrophil elastase is strongly reduced. It is caused by A1AT deficient variants but also by “Null” *SERPINA1* alleles which have no proteic expression at all [2, 3]. In the second one, the liver disease is mediated by the aggregation of misfolded A1AT molecules into ordered polymers that become sequestered in characteristic inclusions within the endoplasmic reticulum (ER) of hepatocytes, thus leading to their apoptosis.

Several regions within the molecule of A1AT control the conformational changes which occur during the process of protease inhibition [4]. One of these, called the breach, is located at the top of β-sheet A where the reactive center loop inserts after cleavage by the proteinase. The importance of this region is reflected the presence of highly conserved residues and by the fact that the most frequent point mutation associated with severe A1ATD (Glu342Lys) is precisely located in the breach. The resulting Z-proteins undergo intra-hepatocyte polymerization causing liver damage and low circulating A1AT concentration promoting development of COPD. Another region located in the middle of the serpin, called the shutter, is also characterized by the presence of conserved residues. It controls the opening of the A-sheet and mutations in that region result in dysfunctional A1AT variants that are associated with a severe A1ATD and hepatic inclusions due to the formation of loop-sheet polymers: Pi Siiyama (Ser53Phe) and Pi Mmalton (Phen52del) [5, 6] (also known as Mnichinan if associated with Gly148Arg). Two other deficient variants in the shutter region, namely Pi S (Glu264Val) [7] and Pi I (Arg39Cys) [8], also induce polymerization but their rates of polymers formation are much slower than for Pi Z, Pi Siiyama and Pi Mmalton, thus leading to a milder plasma deficiency without liver disease.

A1ATD is present in all racial subgroups worldwide but with striking differences in the calculated prevalence of the PiZ phenotype. For instance, this prevalence is about 1 out of 7500 individuals in Southern Europe (Spain, Portugal, France and Italy) [9], 1 out of 2700 in Northern Europe and 1 out of 18,000 in central Europe [10]. As stated by de Serres, A1ATD is not a rare condition but a condition that is rarely diagnosed [11]. As a matter of fact, it is estimated that in Europe and in the USA, less than 10 % of the expected individuals with Pi Z phenotype are identified [12]. Because the condition is thought to be rare, physicians are not familiar with the symptoms which are often categorized as common COPD without addressing the possibility of A1ATD. Another reason might be the costs of the tests which are expensive and not always easily available. Luckily, since a few years, as there is a growing interest in AATD, rarer variants are more often identified [1, 13] and, among them, some are polymerogenic and can be associated with a mildly or severely decreased concentration of A1AT that may lead to emphysema and/or liver disease: Pi Queen’s (Lys154Asn), Pi Pbrescia (Gly225Arg), Pi Mpisa (lys259Ile), Pi King’s (His334Asp), Pi Etaurisano (Lys368Glu), Pi Yorzinuovi (Pro391His), Pi Wurzburg (Pro369Ser) and Pi Mheerlen (Pro369Leu).

The currently used algorithm for the laboratory diagnosis of A1ATD combines quantification, phenotyping and genotyping [14]. Quantification is mostly realized using immunological procedures such as immunoturbidimetry or immunonephelometry whereas phenotyping is usually assessed with isoelectric focusing (IEF) in a narrow pH gradient on polyacrylamide or agarose gels. While the American Thoracic Society/European Respiratory Society stated it as the gold standard for the A1AT ‘genetic testing’ in their 2003 report [15], IEF presents some drawbacks with Q0 alleles and rare deficient variants that imply to resort to genotyping. Unfortunately, the most common genetic techniques which rely on a specific search for the Pi Z and Pi S alleles by restriction length fragment polymorphism (RFLP) [16] or fluorescence resonance energy transfer (FRET) [17] do not also identify Q0 alleles and rare deficient variants. Thus, they should always be associated with the quantification of the protein, otherwise a non S/non Z patient might be considered as having a non pathological phenotype while he might be Pi Mmalton/Null for instance. So, when a low concentration of A1AT cannot be attributed to a Pi ZZ genotype, the direct Sanger sequencing of the entire *SERPINA1* gene and/or specific PCR based methods for some rare or Null variants must be performed until an explanation can be found [18]. A few Q0 alleles caused by the deletion of one or more of the *SERPINA1* gene coding exons have also been described [19].

## Methods

In the present paper, we describe four cases in which the Pi Mmalton allele was first biologically and/or clinically suspected before being confirmed by Sanger DNA sequencing. Interestingly, 3 different genotypic situations are explored: Mmalton/Mmalton (patient 1), Mmalton/Z (patients 2 and 3) and M/Mmalton (patient 4). All four probands signed a written agreement for genetic analysis according to the Helsinki declaration.

### Case 1: Mmalton/Mmalton

A 80-year-old woman of Algerian origin was admitted to hospital for the treatment of an acute and painful fracture of the third lumbar vertebra. A computed tomography scan (CTS) was performed for this fracture and revealed a dysmorphic and enlarged liver with signs of portal hypertension (splenomegaly, collateral veins). Her past medical history was significant for obesity, vertebral osteoporosis, monoclonal gammapathy of undetermined significance (IgG kappa) and some bronchitis episodes in the past 2 years. She had neither history of liver disease nor alcohol, tobacco or illicit drug abuse. Her medical examination revealed the absence of ascites, peripheral edema, jaundice and asterixis. Laboratory tests showed abnormal liver function tests: alkaline phosphatase, 382 UI/L (reference interval, 45–117 UI/L); gamma-glutamyl-transpeptidase, 299 UI/L (reference interval, 5–55 UI/L) aspartate aminotransferase, 56 UI/L (reference interval, 15–37 UI/L); alanine aminotransferase, 25 UI/L (reference interval, 12–78 UI/L); total serum bilirubin, 0.94 mg/dL (reference interval, 0.176–1.0 mg/dL). Serum albumin was decreased, 29 g/L (reference interval, 35–50 g/L). Blood platelets count was low, 143 × 10^9^/L (normal range, 150–400 × 10^9^/L). INR was 1.2 and serum creatinin was 47 μmol/L. Liver stiffness measurement by transient elastography (Fibroscan®) was pathologic with an increased value of 21.3 kPa in favor of underlying cirrhosis (normal range <7–8 kPa). A gastroscopy revealed grade 2 esophageal varices with red spots which were treated by band ligation. The serological tests for viral hepatitis and autoimmune hepatitis were negative. Serum cholesterol, triglycerides, ceruloplasmin and ferritin levels were normal.

**Fig. 1:**
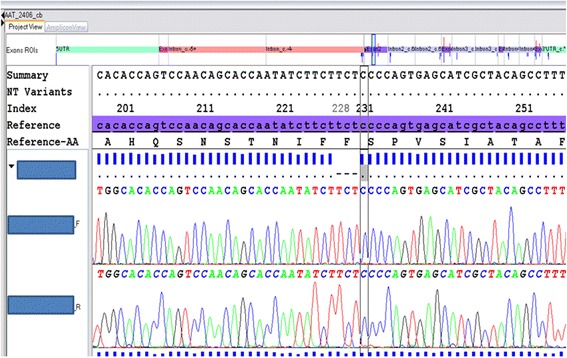
Part of DNA sequencing of the *SERPINA1* gene, showing the deletion of three nucleotides in exon 2, codon 52 (TTC > 0) at the homozygous state (a Phe amino acid residue is deleted). HGVS nomenclature: SERPINA1:c.154_156del; p. Phe52del

**Fig. 2:**
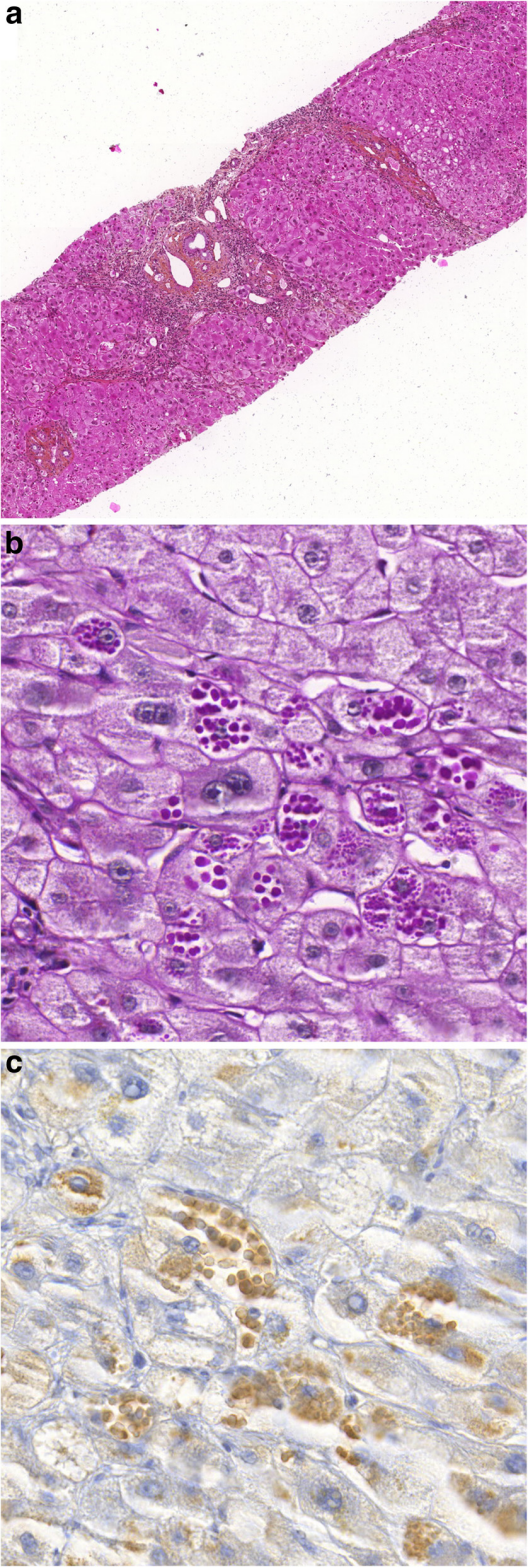
Liver tissue was obtained by percutaneous liver biopsy of the proband. **a** Hematein Phloxin Safran (HPS) coloration showing collagen fibrosis around hepatocytes with numerous round eosinophilic cytoplasmic inclusions; (**b**) PAS staining with diastase digestion showing abundant hyaline globules containing A1AT accumulated within periportal hepatocytes; (**c**) Immunohistochemical staining using a monoclonal antibody to A1AT

Protein serum electrophoresis (Capillarys®, Sebia) revealed a marked decrease of the α_1_-globulin fraction strongly suggesting an A1AT deficiency. The low serum A1AT level determined by immuno-nephelometry (0.34 g/L, reference interval: 1.0–1.8 g/L) and the isoelectric pattern with faint bands slightly more cathodal than M2 bands were very suggestive of a Pi Mmalton allele at the homozygous state. To confirm this diagnosis, we performed the Sanger DNA sequencing of the entire *SERPINA1* gene with the ABI Prism 3130XL (Applied) as previously described [20]. Sequence analysis (Fig. 1) revealed the presence, at the homozygous state, of a deletion of the three nucleotides TTC in codon 52 (exon 2) corresponding to the Mmalton allele (HGVS nomenclature: *SERPINA1*:c.154_156del; p. Phe52del). This deletion was on a M2 background since it was associated with the homozygous c.374G > A and c.1200A > C single nucleotide polymorphisms, thus defining the Pi Mmalton allele (the Phe52deletion on a M1 background has been called Pi Palermo). A percutaneous liver biopsy revealed micronodular cirrhosis, without steatosis but with periodic acid-Schiff (PAS)-positive acidophilic, diastase-resistant bodies in the cytoplasm of the hepatocytes. Immunohistochemical staining using a monoclonal antibody to A1AT was positive (Fig. 2). According to Child-Pugh classification and MELD score, the cirrhosis was Child-Pugh B7, MELD 9. With regards to the lung, a thoracic CT scan revealed few lesions of bronchiectasis but without lesions of emphysema and the pulmonary function tests were within the normal range.

### Cases 2 & 3: Mmalton/Z

A 41-year-old man of French Caucasian origin was hospitalized for severe dyspnea. According to his medical records, he suffered from COPD, chronic emphysema and had presented with many bronchitis episodes in his youth, when he used to be a frequent smoker. A 37-year-old woman of French Caucasian origin was seen in medical consultation for asthma and bronchiectasis. Her medical history revealed allergic asthma equilibrated by Symbicort® but no sign of COPD.

In both cases, the biochemical A1AT analysis revealed a very low serum level (0.33 and 0.22 g/L, respectively) and a Pi Z phenotype at IEF but a genetic confirmation performed with a RFLP method specific for the Pi Z and Pi S alleles identified the Pi Z allele at the heterozygous state only. This discrepancy could be solved by Sanger DNA sequencing which revealed a Pi Mmalton/Pi Z compound heterozygosity. Hepatic function tests were then performed for both patients but they appeared normal.

### *Case 4*: M/Mmalton

A 57-year-old woman of Moroccan origin suffering from an end-stage kidney disease was admitted for pre-kidney transplant investigations. Her medical story revealed diabetes with various peripheral vascular complications but neither pulmonary nor hepatic dysfunction. Serum electrophoresis revealed a double α1-globulin peak, suggesting the presence of a Pi A1AT protein variant in the heterozygous state. The A1AT IEF phenotype was noted Pi M1/Pi M2 but because of an abnormally low serum concentration (69 mg/dL), DNA sequencing was performed and revealed a Pi M1/Pi Mmalton genotype.

## Results and discussion

**Fig. 3:**
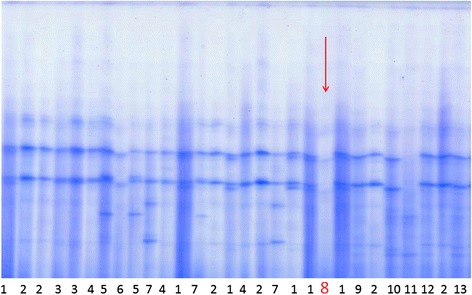
Isoelectric focusing gel showing a pure Pi Mmalton profile among the main other classical A1AT Pi profiles: 1:M1M2; 2:M1; 3:M1M4; 4:M1M3; 5:M1V; 6:M1X; 7: M1S; 8: Mmalton; 9:M1Z; 10:M2Z; 11:Z; 12:M1Z et 13:M3Z. We can particularly notice the very weak expression of the bands in the Mmalton lane compared to others lanes. This ‘pure’ Mmalton profile can correspond to a Mmalton homozygosity or a Mmalton/Null compound heterozygosity

The Pi Mmalton variant was first discovered by Cox in 1976 [21]. Maybe because of a similar genetic background with the normal Pi M2 allele, it is characterized by a slightly more cathodal mobility than M2 on IEF gels. At the heterozygous state, it thus may be confounded with an M protein [22] but such a confusion can easily be prevented by A1AT quantification since a Pi MM genotype is much of the time not compatible with an A1AT serum value below 1 g/L which is observed in that case. The situation is more complex and the Pi Mmalton variant can easily be missed when it is associated with a Pi Z protein [23] or with any other rare A1AT allele [24]. At the homozygous state, the Pi Mmalton protein is clearly visible on the IEF gel (Fig. 3) and this situation is phenotypically undistinguishable from a Null/Pi Mmalton compound heterozygosity. A Sanger direct sequencing is thus required for the differential diagnosis which is important because of the liver dominant negative effect of the Pi Mmalton variant. The exact pathogenesis is incompletely known but it seems that apoptosis is more pronounced in hepatocytes with greater levels of insoluble polymerized/aggregated forms of A1AT that accumulate in the ER, the soluble forms being more specifically degraded by the proteasome [25]. The first cases of Pi Mmalton homozygosity were reported in 1987 by Reidl (association of emphysema, cirrhosis, and hepatocellular carcinoma in a 63-year old white man) [26] and in 1989 by Curiel (47-year-old male patient with a 20-pack-year smoking history suffering from emphysema and hepatic inflammation) [27]. Up to these case reports, the Pi Mmalton protein was thought to be associated with emphysema only. In case 1, because of the paucity of the known proband’s medical records, it was not possible to determine when the hepatic disease appeared but the fibrosis had certainly begun many years ago since a quite advanced cirrhosis was present at the time of diagnosis. The fact that no pulmonary disease was observed is probably explained by the non-smoker profile of the proband.

Since then, with the growing interest in A1ATD, the improvement in the genetic techniques and the knowledge that there were deficient variants others than S and Z, some studies were devoted to the prevalence and clinical phenotypes of subjects carrying rare deficient variants. It appeared among other things that the Pi Mmalton variant was not as rare as previously thought: indeed it seems that it is the most frequent deficient variant in Sardinia [28] and Tunisia [29] and the third one (after Pi S and PiZ) in Spain [30]. It is noteworthy that 2 of our 4 reported patients were issued from the Maghreb countries. Since there is a large maghrebian community in France, it would be important to set a strategy of A1ATD diagnosis allowing not to miss the possibility of a Pi Mmalton allele. It becomes also necessary to document the clinical phenotypes of homozygous and heterozygous patients. Ferrarotti [28] reported that liver disease was detected in 13 % of subjects that were either homozygous or heterozygous for the PiMmalton variant compared to 11 % in Pi ZZ patients. The Mmalton/Z and M/Mmalton have also been frequently observed in the literature and they were associated with very different clinical states. Cox made the first description of the PiMmalton variant in a family exhibiting Pi MmaltonZ and Pi MmaltonM phenotypes without any liver or lung involvement but all members were below 30 years of age [21]. On the other hand, Canva et al. described a case of a Mmalton/M 59-year-old woman with end-stage liver disease, despite no history of hepatitis, alcohol abuse or childhood liver disease [31]. Between these two extremities, most of the described M/Mmalton patients had normal pulmonary and liver functions while the Mmalton/Z genotype was often, but not always, associated with a high risk of developing emphysema, particularly in smokers [26, 32, 33].

The case-reports presented in the present study are in accordance with that: patient 2, who was a smoker, suffered from COPD while patient 3, a non-smoker, had no major pulmonary sign. For both patients, their young age at the time of observation has probably prevented them from any hepatic disorder. Obviously, exposure of environmental factors is also of major importance in the apparition of the lung and liver diseases for Mmalton/Z patients. It is very well-known and evident in the former (tobacco and emphysema) but it is true as well in the latter since any drug or toxic (ethanol), that negatively regulates autophagy, worsens liver apoptosis. Conversely, recent studies have shown that carbamazepin, a drug that enhances autophagy, decreases the hepatic fibrosis in a mouse model of A1ATD [34]. Thus, clear genotype-phenotype relationships deficiency in A1ATD would necessitate large cohorts to withdraw the environmental bias.

## Conclusions

As stated above, the Pi Mmalton variant is very important to diagnose and should never be missed once an A1AT examination has been prescribed by a physician. To do so, medical biologists implied in the diagnostics of AATD should at least be aware of the pattern of migration of the PiMmalton protein and be very cautious in front of any discrepancy with the A1AT serum level. If a genetic analysis has to be done, Sanger direct sequencing is highly recommended to ensure a 100 % sensibility whatever the *SERPINA1* allele.

Our study confirms the great clinical heterogeneity of the Mmalton/Z genotypes but, obviously, the exposure of environmental factors complexifies much the interpretation for such small series of patients. Very interestingly, a rapid and reliable genetic method for the detection of the Pi Mmalton allele on whole blood, serum and dried blood spot has just been published [35]. Using it in a large-scale screening would allow to obtain information about the real prevalence of the Pi Mmalton allele and to constitute large series of patients for a proper phenotype-genotype relationship.

### Consent to publish

The four probands were asked if their medical data could be anonymously used for a scientific publication and they all agreed.

## Abbreviations

A1AT: Alpha 1 antitrypsin
A1ATD: Alpha 1 antitrypsin deficiency
COPD: Chronic obstructive pulmonary disease
ER: Endoplasmic reticulum
IEF: Isoelectric focalization
CT: Computed tomography
MELD: Model for end-stage liver disease
PAS: Periodic acid-Schiff
RFLP: Restriction length fragment polymorphism
DNA: Desoxyribo nucleic acid
FRET: Fluorescence resonance energy transfer
